# Is the diatom sex clock a clock?

**DOI:** 10.1098/rsif.2021.0146

**Published:** 2021-06-16

**Authors:** Thomas Fuhrmann-Lieker, Nico Kubetschek, Jonas Ziebarth, Roland Klassen, Werner Seiler

**Affiliations:** ^1^ Physical Chemistry of Nanomaterials, Institute of Chemistry and Center for Interdisciplinary Nanostructure Science and Technology, University of Kassel, 34109 Kassel, Germany; ^2^ Microbiology, Institute of Biology and Center for Interdisciplinary Nanostructure Science and Technology, University of Kassel, 34109 Kassel, Germany; ^3^ Algorithmic Algebra and Discrete Mathematics, Institute of Mathematics, Faculty of Mathematics and Natural Sciences, University of Kassel, 34109 Kassel, Germany

**Keywords:** diatoms, oscillations, clocks, discrete models, matrix models

## Abstract

The unique life cycle of diatoms with continuous decreasing and restoration of the cell size leads to periodic fluctuations in cell size distribution and has been regarded as a multi-annual clock. To understand the long-term behaviour of a population analytically, generic mathematical models are investigated algebraically and numerically for their capability to describe periodic oscillations. Whereas the generally accepted simple concepts for the proliferation dynamics do not sustain oscillating behaviour owing to broadening of the size distribution, simulations show that a proposed limited lifetime of a newly synthesized cell wall slows down the relaxation towards a time-invariant equilibrium state to the order of a hundred thousand generations. In combination with seasonal perturbation events, the proliferation scheme with limited lifetime is able to explain long-lasting rhythms that are characteristic for diatom population dynamics. The life cycle thus resembles a pendulum clock that has to be wound up from time to time by seasonal perturbations rather than an oscillator represented by a limit cycle.

## 1. Introduction

Phytoplankton plays an enormous role in the sequestration of carbon dioxide from the atmosphere [1,2]. From the estimated 60 Gt of carbon fixation per year in the oceans, 26 Gt are attributed to diatoms, both values being maximum estimates [3]. Additional contributions come from freshwater in which diatoms are also abundant, leading to a total share of 20–25% of primary production worldwide. However, productivity is subjected to periodic fluctuations in the form of the so-called algal blooms [4,5]. Whereas a major part of these fluctuations can be attributed to seasonal changes in nutrient availability, temperature and photoperiod, diatoms possess an intrinsic oscillation mechanism in their population due to their peculiar life cycle that interferes with external conditions. Thus, understanding the population dynamics of diatoms helps in understanding global carbon cycles.

In 1984, William M. Lewis [6] of the University of Colorado described the unique life cycle of diatoms (Bacillariophyta) as a ‘diatom sex clock’. Briefly, it comprises two alternating phases, a long mitotic size diminution phase and a short sexual size restoration phase. The uniqueness in size reduction and restoration is a consequence of the specific cell wall structure consisting of two biomineralized silica halves with different size (*epitheca* and *hypotheca*) that are assembled like a Petri dish. Upon cell division, each half becomes an *epitheca* of the next generation, in which a new *hypotheca* is synthesized. By this mechanism, the mean cell size of a population decreases from generation to generation, and the distribution of sizes becomes larger (MacDonald–Pfitzer rule) [7,8]. Obviously, this process cannot proceed forever, so later generations undergo sexual reproduction and form haploid gametes which eventually fuse to diploid auxospores. Within these auxospores, new large initial cells are produced, starting the cycle again. The variety of sexual mechanisms is large [9,10]. In some cases, auxosporulation can occur within one single cell (*uniparental*), whereas, in other cases, gametes from different cells have to recombine (*biparental*). Within these two main types, several variants are observed. In uniparental auxosporulation such as *automixis*, sexuality is reduced to some degree. Biparental (*allomictic*) auxosporulation can occur in *homothallic* species (gametes of both sexes are formed within a single clonal strain) or *heterothallic* species (strains of different mating types are required) [10]. Von Stosch was able to show by experiments using artificial alteration of size that the threshold for sexual reproduction is determined by cell size rather than by age [11,12]. Therefore, a specific size range of cells that are capable of sexual reproduction can be assumed for each individual species. If the cell is larger than an upper sexual size threshold, auxosporulation is blocked [10,13]. In some cases, also a lower size limit for auxosporulation exists; the life cycle is then referred to as ‘closed’. In any case, there is a minimum size until which the cells are viable. Together with the maximum size of the initial cells, this defines the possible range for the cell size. It should be noted that while the size reduction–restitution mechanism is widespread and distinguishes diatoms from other taxa, exceptions are described showing no decrease in size during the vegetative phase or even vegetative enlargement in order to produce larger cells [9,14]. However, by the standard mechanism, as Lewis pointed out, the diatom life cycle (‘sex clock’ or generation clock) defines its own rhythm independent from environmental constraints. Cell size reduction–restitution cycles that can last many years in natural environments apparently support this hypothesis [15].

There is a caveat, however, for taking the diatom sex clock simply as a periodic process, and that is caused by the broadening of the cell size distribution. If the size distribution broadens continuously and larger cells are maintained in the system, it is difficult to close the cycle and return to a previous state without any additional mechanisms that counteract this broadening. While this is certainly a challenge to the clock concept, field observations of diatom populations over many years confirm periodic fluctuations with the proposed periodicity of the life cycle [15–17]. Mathematical models may help in understanding the possible mechanisms that cause a deviation from the simple step-by-step size reduction–restitution scheme. Simulations have been made with a large set of parameters aimed at matching the experimental observations, notably in the work of Schwarz *et al*. [18], D'Alelio *et al*. [17] and Hense & Beckmann [19]. Schwarz *et al.* [18] fitted experimental data for *Pseudo-nitzschia delicatissima* with a continuous-time Markov chain model and investigated the stationary distribution without oscillations. D'Alelio *et al*. [17] modelled the cell size dynamics of *Pseudo-nitzschia multistriata* over 11 years in an eight-parameter model with polynomial differential equations. The 14-parameter model of Hense & Beckmann [19] took the environment into account by modelling vertical migration in a water column. In their study, multimodal size distributions were attributed to intra- and interspecific competition as a way to overcome the broadening of the size distribution. With this contribution, we address this issue from a different perspective and ask under which circumstances real periodicity can be obtained in a discrete minimum model derived from the generation-resolved MacDonald–Pfitzer scheme and what are the obstacles and conditions for maintaining the clock over many periods.

## 2. Material and methods

We describe the state of a diatom population in the basic model (referred to as *basic linear model*) as a vector ***x*** of size *n*, in which each element represents the number of individuals of a distinct size class per volume. The order of sizes is chosen such that higher indices characterize larger sizes and we start with the index *i* = 0 for the smallest size, leaving for the largest size (i.e. the initial cells) the index *n* − 1. We adopt this enumeration convention starting with 0 (rather than 1) in order to be compatible with the programming language and algorithms used in the simulations.

Using different stages for a single species—a concept that we transfer here to the diatom life cycle—is known as the Leslie model [20] in biomathematical literature. Briefly, in the Leslie model, a (female) animal population is divided into different age classes, typically three, with separate transition rates to the next generation. Whereas the Leslie model and other models for population dynamics typically treat time as a continuous variable, we account for the special proliferation mechanism with discrete size steps at discrete time points and model the system as a discrete dynamical system [21]. This means that we determine the population state at discrete points in time *t,* defining distinct generations in accordance with the MacDonald–Pfitzer rule. The transition from one generation to the next is thus expressed by a propagation matrix ***P*** applied to the current population state

2.1 $$x_{t+1}=Px_{t}.$$

The elements of this matrix are determined by the two states in the diatom life cycle. The vegetative phase is represented by the diagonal elements denoting daughters derived from the epitheca, and the upper side diagonal denoting daughters from the hypotheca. The probability of successful division and survival to the next generation is given as parameter *p*. In order to treat a possible bias between the two daughters in this probability, we multiply the diagonal elements by *β*. For size classes that are capable of auxosporulation, a different factor for remaining *α* is assigned. The sexual phase is represented by a parameter *s*, which defines the probability for the lower size classes to form auxospores and initial cells in the next step. With *s* in the lower left corner of the propagation matrix, the life cycle is closed. In order to be able to extract *p* from the propagation matrix and to simplify the resulting analytical expressions for general *n*, we write *s = σ^n^p* and conveniently use *σ* instead of *s* as the parameter. For reference, all parameters are compiled in table 1.

**Table 1.** Parameters used in the models.

parameter	meaning	model
*N*	number of size classes	all models
*i*	index for size class, 0 ≤ *i* < *n*	all models
*p*	survival probability of theca	all models
*s*	probability of auxosporulation	all models
*σ*	(*s/p*)^1/*n*^	all models
*α*	survival factor of smallest cell	all models
*β*	asymmetry factor between daughters	all models
*t*	generation as time scale	all models
*γ*	inverse carrying capacity	saturation models, ageing models
*m*	number of age classes	ageing models
*j*	index for age class, 0 ≤ *j* < *m*	ageing models
*z*	period of a zeitgeber in generations	zeitgeber models

For simplicity and parameter reduction, we assume that only the smallest size class can undergo auxosporulation, thus contracting the corresponding size range lower than the upper threshold for auxosporulation to one single class. The propagation matrix of the basic linear model is thus given as

2.2 $$\begin{array}{cc} P=\; & \left(\begin{array}{ccccccc} \alpha p\; & p\; & 0\; & \ldots \; & 0\; & 0\; & 0\;\\ 0\; & \beta p\; & p\; & \ldots \; & 0\; & 0\; & 0\;\\ 0\; & 0\; & \beta p\; & \ldots \; & 0\; & 0\; & 0\;\\ \ldots \; & \ldots \; & \ldots \; & \ldots \; & \ldots \; & \ldots \; & \ldots \;\\ 0\; & 0\; & 0\; & \ldots \; & \beta p\; & p\; & 0\;\\ 0\; & 0\; & 0\; & \ldots \; & 0\; & \beta p\; & p\;\\ s\; & 0\; & 0\; & \ldots \; & 0\; & 0\; & \beta p\; \end{array}\right)\;\\ =p\; & \left(\begin{array}{ccccccc} \alpha \; & 1\; & 0\; & \ldots \; & 0\; & 0\; & 0\;\\ 0\; & \beta \; & 1\; & \ldots \; & 0\; & 0\; & 0\;\\ 0\; & 0\; & \beta \; & \ldots \; & 0\; & 0\; & 0\;\\ \ldots \; & \ldots \; & \ldots \; & \ldots \; & \ldots \; & \ldots \; & \ldots \;\\ 0\; & 0\; & 0\; & \ldots \; & \beta \; & 1\; & 0\;\\ 0\; & 0\; & 0\; & \ldots \; & 0\; & \beta \; & 1\;\\ \sigma^{n}\; & 0\; & 0\; & \ldots \; & 0\; & 0\; & \beta \; \end{array}\right)\; \end{array}$$

Considering a whole size range and introducing an additional parameter as the upper size threshold or even a distribution of initial sizes is possible and would give additional entries in the matrix. The corresponding expressions are shown in the electronic supplementary material. We can safely ignore all classes smaller than a possible lower size threshold for auxosporulation, since they will not contribute further to the cycle and eventually die out.

In the basic linear model, the auxosporulation probability is proportional to the number of possible parent cells.

We note that a generation-resolved discrete matrix model without implementation of a sexual phase was used by Terzieva & Terziev [22], whereas other previous models were based on differential equations, the one of Schwarz *et al*. [18] also being linear and others using polynomial or further nonlinear expressions [17,19].

Four classes of variations of the basic linear model are considered.

First, possible delays of one generation caused by short resting phases in epitheca, hypotheca or auxospores are treated. They could be modelled with second-order difference equations (delay difference equations [23])

2.3 $$x_{t+1}=P_{0}x_{t}+P_{1}x_{t-1},$$

but we rewrite this expression using a single matrix and state vectors of double size containing both ***x****_t_* and ***x****_t−_*_1_ (see electronic supplementary material, S2). These variations are called *delay models.* In the *Müller delay model* the smaller daughter is delayed by one generation, whereas in the *Laney delay model* the larger daughter is delayed by one generation.

Second, a nonlinearity is introduced (*nonlinear models*). A biparental scheme for sexual reproduction was modelled by

2.4 $$x_{n-1,t+1}=\frac{sx_{0,t}^{2}}{\left(1+2sx_{0,t}\right)}\;(\mathrm{biparental}\;\mathrm{nonlinear}\;\mathrm{model}),$$

instead of

2.5 $$x_{n-1,t+1}=sx_{0,t},$$

as in the basic linear model.

In an independent nonlinear model, overpopulation was avoided by introducing a saturation limit for the population according to a Ricker function [24]. Extending the Ricker function to multiple size classes, *p* drops exponentially with the total number of cells according to

2.6 $$\;p=p_{0}e^{-\gamma {\left(\sum_{i}x_{i}\right)}_{t}}\;(\mathrm{saturation}\;\mathrm{nonlinear}\;\mathrm{model}).$$

Saturation nonlinearity was also used in the following two classes of models.

Third, for simulating a finite lifespan (*ageing model*), we coded the age of the *epitheca* of a cell as an additional dimension of size *m*, representing *m* different age classes (index *j* with 0 ≤ *j* < *m*). Hence, the state vector was replaced formally by a matrix of size *n* × *m,* and the propagation matrix formally by a tensor of fourth rank. In the analytical treatment (electronic supplementary material, S3), the state matrix was rewritten to a simple vector as in the case of the delay model, so that ***P*** could still be treated as a matrix. For the computational treatment, a fast algorithm was devised (electronic supplementary material, S5), taking advantage of the fact that only some elements differ from zero. The new population was then calculated as the sum of only two contributions: (i) cells from the epithecae, formed by shifting the previous population matrix *to the right* (increase of age by 1), removing aged cells and correcting the remaining ones by the appropriate factors *α p* and *β p*; and (ii) cells from the hypothecae, formed by shifting the previous population matrix *upwards* (decrease in size). The matrix multiplication was, therefore, reduced to two ‘roll’ commands. Additionally, new large cells from auxospores were considered. This computational trick had been applied in most simulations, also in implementations of the other models in which only the second roll was needed.

Fourth, *p* or *s* are made variable in order to account for seasonal changes in growth and auxospore formation (*zeitgeber models*). A sinusoidal variation between a maximum and minimum value was assumed as the basic mode. In the *p-zeitgeber model*, *p* is varied, whereas in the *s-zeitgeber model*, *σ* is varied.

For analysing all investigated cases, we used a combination of two methods, an analytical one and a computational one. Whenever possible, we performed a mathematical analysis in order to find the contributing eigenvalues and eigenvectors. If the eigenvalues *λ_k_* and corresponding eigenvectors *ξ_k_* of ***P*** are known, then, for arbitrary initial conditions, the temporal evolution of the population after *t* generations can be predicted as a sum of distinct relaxation modes according to

2.7 $$x_{t}=P^{t}x_{0}=\sum_{k}\lambda_{k}^{t}c_{k}\xi_{k}.$$

Here, the coefficients *c*_i_ denote the decomposition of the initial vector ***x*_0_** into contributing eigenvectors.

This analytical treatment is supplemented by computer simulations, especially in the cases where analytical expressions for the eigenvalues are not easily possible (saturation models, ageing model, zeitgeber models). Programs containing the different model variations were written in Python. Source codes are provided in electronic supplementary material, S5 and S7. In the computer simulations, we sampled as suitable measures for the population state at a certain point in time:

the total number of cells

2.8 $$N=\sum_{i=0}^{n-1}x_{i},$$

the number of formed auxospores

2.9 $$A=sx_{0},$$

the mean size (first moment of the distribution)

2.10 $$M_{1}=\frac{1}{N}\sum_{i}x_{i}i$$

and the variance $V=M_{2}-M_{1}^{2}$, derived from the second moment

2.11 $$M_{2}=\frac{1}{N}\sum_{i}x_{i}i^{2}.$$

## 3. Results and discussion

Using different models, it is analysed under which assumptions oscillations of the population state are obtained, and whether these oscillations can be self-sustained or need external input. A sustained or driven oscillation with long periods would constitute a sex clock in the meaning attributed by Lewis to the diatom life cycle. We start with a linear model*,* then this model is extended systematically in order to demonstrate which changes have to be made in order to favour oscillatory behaviour. In contrast with multi-parameter models aimed at ecosystem simulations for matching field data [17–19], the number of parameters is kept as small as possible to show the principal logic behind the mechanisms. The systematic approach of extracting analytical information from a linear model extended to delay and ageing processes, while being able to implement nonlinearities and zeitgeber by varying the parameters of the model computationally is a unique feature that distinguishes our approach from previous reports.

### 3.1. Basic linear model

The basic linear model represents the original MacDonald–Pfitzer rule. With this model, we can rigorously answer the question why the model needs a modification in order to explain sustained oscillations in the population distribution. The MacDonald–Pfitzer rule refers to distinct generations, thus the application of a discrete model with distinct time steps is well justified. We note that diatom cultures can even be synchronized (with the help of nutrient starvation or blue light [25]), which would allow an experimental verification of generation-resolved models. However, not enough data are available at the moment.

As explained in the Material and methods section, the population state consists of the number of cells for each size. In real species, up to 500 different sizes can be achieved with an average generation time of one week, resulting in life cycles of several years [15]. For understanding the generic behaviour of the model, the exact number of sizes is not important, though.

The main parameters used in the model are the vegetative proliferation probability *p*, the sexual proliferation probability *s,* an asymmetry factor between the daughter cells in the vegetative phase *β* and a survival factor for the smallest cells *α* (table 1)*.* In our basis model, all sizes that are capable of auxosporulation are contracted into this single size class. This approach seems to be a rough approximation, but we will show at the end of the section that the main conclusions also hold if we consider a distribution of sexual stages or initial cell sizes. By construction, the linear model represents uniparental sexual schemes, but biparental schemes are covered as well in the limit of high cell density (see §3.3).

**Figure 1.**
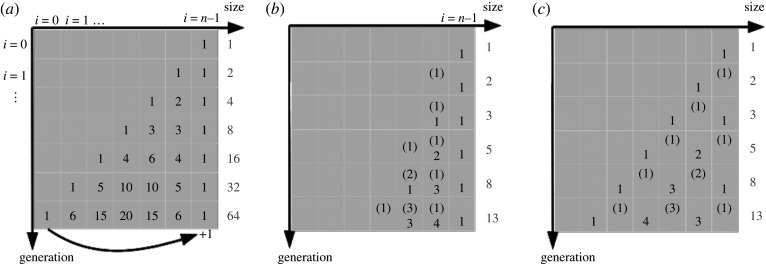
Three different models for the proliferation mechanism of diatoms: (*a*) simple binary fission (basic linear model) and (*b*) binary fission with a delay of one generation for the hypothecal daughter (Müller delay model). (*c*) Binary fission with a delay of one generation for the epithecal daughter (Laney delay model). Cells that cannot divide in the next generation are denoted with brackets. The total number of cells is counted in the columns on the right-hand side (grey numbers).

The ‘ideal’ case in which there are no losses of cells and doubling in the vegetative phase is characterized by the parameters *p = β* = 1, *α =* 0 and *s* = 1. With one initial cell of largest size as the usual initial condition, the occupation numbers of the different size classes follow directly from Pascal's triangle (figure 1*a*) with a total number of 2*^t^* cells after *t* generations, up to the point where the smallest class is reached and new initial cells via auxosporulation are generated.

**Figure 2.**
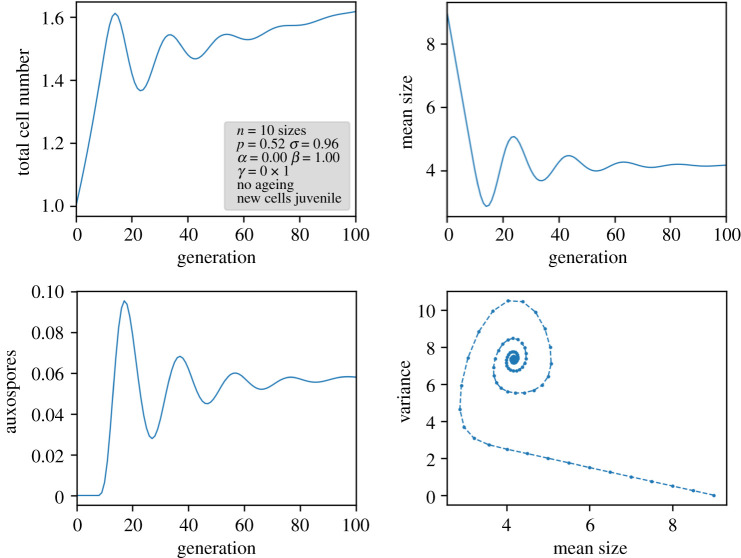
Results of the linear model with parameters near to a true steady state, characterized by a constant total cell number (per unit volume) as well as a constant population distribution.

The number of cells would be invariant if only half of the cells in the vegetative phase survived, i.e. *p* = 0.5, and other parameters were unchanged. For intermediate values of *p*, there is a value of *s* that in the long term balances the growth in the vegetative phase by loss in the sexual phase. In figure 2, a typical result is plotted for the linear model with parameters near to this steady state. It reflects the general behaviour obtained for this model: oscillations in the output variables occur, but they decay and approach an equilibrium in the long term.

This behaviour can be attributed to the continuous broadening of the size distribution which prevents a periodic transition of a narrow size peak through all size classes. In physical terms, such a transient wave without broadening would be described as a ‘soliton’.

Next, it is shown how the problem of the relaxation behaviour can be transformed into mathematical language. Doing this, we can prove rigorously that oscillations in the linear model will always decay regardless of the parameters, and which concepts for the mechanism will not be able to change this behaviour. The temporal evolution of the system is described by equation (2.5) and thus depends on the eigenvalues of the matrix ***P***. The leading (dominant) eigenvalue *λ*_max_, i.e. the one with the largest absolute value, and its corresponding eigenvector will dominate the population in the long term, since contributions from all other eigenvalues are decaying faster (or not growing so fast). Oscillations in the population will be indicated by complex eigenvalues or negative real eigenvalues that indicate a cycle of two generations. Therefore, if the dominant eigenvalue *is not real positive*, we will find sustained oscillations. In all other cases, intermediate oscillations will decay in the long term.

An answer to whether sustained oscillations are possible or not can be given quite elegantly by the application of the Perron–Frobenius theorem [26,27]. It states that, for non-negative irreducible matrices, the dominant eigenvector *is always* positive. Matrix ***P*** is non-negative because none of the matrix elements has a negative sign, and irreducible because it reflects a cyclic mapping of one row index in the state vector to the next lower one and therefore exhibits a strong connectivity (for a mathematical proof, see electronic supplementary material, S1). However, decaying oscillations exist and we can analyse the decay parameters represented by the non-leading eigenvalues.

For *α = β*, the eigenvalues of ***P*** can be easily calculated for general dimension *n* (see electronic supplementary material, S1)*.* They are evenly distributed in the complex plane on a circle around *p β* with radius *r* = *p σ.* The dominant eigenvalue is located on the real axis. For odd *n*, this is the only real eigenvalue. For even *n*, there is a second one at *p*(*β* − *σ*). It should be remarked that eigenvectors with negative eigenvalues may contribute but can never dominate, since, for any non-negative initial vector, a change of sign is prevented by the non-negative values in the matrix. An important case for the dominating eigenvalue is *λ*_max_ = *p*(*β +σ*) = 1, which represents a stable population with zero growth. For each combination of *β* and *σ*, there is exactly one corresponding value of *p* that leads to this condition. The number of cells is maintained with the eigenvalue of +1 and, owing to the symmetry, the eigenvector for this special case shows an equal distribution among all size classes. If *σ* < 1, the dominant eigenvector represents an exponentially falling distribution from smallest to largest sizes, depending solely on *σ* irrespective of the value for *β*. The other eigenvalues with non-zero imaginary part describe oscillations. Since each trajectory starting from a certain initial state is a superposition of *n* possible relaxation modes, these oscillations are superposed to a decreasing, increasing or stable total population. Since the dominant eigenvector is always present in an initial vector with non-negative occupations, the contributions of these oscillations vanish in the long term.

The period *T* of the oscillations can be readily predicted. The eigenvalues have to be applied *T* times to reach their original phase, so *T* is given by 360° divided by the angle of the eigenvalue to the real axis in the complex plane. Since the *n* eigenvalues are distributed evenly on a circle displaced from the origin by *p β*, this angle is smaller than 360°/*n*. Thus, *T* is larger than *n*.

In the other limit for *α*, i.e. *α* = 0, the situation is more complex and an analytical solution cannot be given easily. However, it is possible to find a relation between *p, β* and *s* such that a steady state with eigenvalue +1 results. To determine the generic case, we used the cofactor expansion method (see electronic supplementary material, S1) to obtain the determinant $\left|P_{n}-\lambda I\right|$ (***I*** is the unity matrix). In short, the characteristic polynomial is given by

3.1 $$|P_{n}-\lambda I|=(\;p\alpha -\lambda ){\left(\;p\beta -\lambda \right)}^{n-1}-{\left(-p\sigma \right)}^{n}.$$

Setting *α* to 0 and *λ* to +1, we can solve the roots for *p* for a given *s*, or calculate *s* for a given *p* via

3.2 $$s={\left(\frac{1-p\beta }{p}\right)}^{n-1}.$$

For a given *s*, the population-balancing value of *p* can be calculated analytically for *n* = 2 and numerically for *n* > 2. Valid solutions are real roots in the interval [0,1]. For *s* = 0.5, *p*-values depend on the dimension as follows: *p =* 2−2√2 ≈ 0.586 (*n* = 3), 0.557 (*n* = 4), 0.543 (*n* = 5) 0.535 (*n* = 6) … . With these specific relations between s and *p*, the number of cells as well as parameters of the distribution are time invariant, thus we obtain a true equilibrium state.

The eigenvalues differ of course, but not too much from the eigenvalues in the case *α = β*, with the same statements on periodicity. Indeed, the simulation in figure 2 reflects the deviation of the period from *n*.

The Perron–Frobenius theorem allows predictions for variations of the model in which other matrix elements are filled. For instance, we can consider a range of auxosporulating sizes below an upper size threshold or also a size distribution of initial cells depending on the parent cell size as observed by Davidovich [28]. The corresponding matrices still have only non-negative entries and retain their connectivity (see electronic supplementary material, S1.3), so, also in this case, occurring oscillations will decay and are not self-sustained.

We can conclude that for a linear system oscillations occur but, in the long term, the trajectory spirals towards a stationary point, the population of which is represented by the dominant eigenvector. Therefore, the simple picture of the life cycle is not able to explain long-term oscillations in the population and size distribution.

### 3.2. Asymmetric delay

In this section, we answer the question of whether a delay in cell division between the daughter cells may cause sustained oscillations. Generally, delay processes are known to favour oscillatory behaviour in various contexts [23,29]. A delay for the smaller daughter by exactly one generation before the next cell division was suggested by Müller [30] in the early days of diatom research after careful studies on chain-forming *Melosira arenaria*. This asymmetry in time is not uncommon for single-celled organisms and is known for other species such as budding yeast [31], in which there is also a difference in size of the two mitotically separated cells. Interestingly, a delay of just the opposite sign was reported by Laney *et al*. [32] for the diatom *Ditylum brightwellii.* In this case, the smaller daughter derived from the previous hypotheca is more likely to divide faster than the one from the epitheca. These contradictory findings certainly express the need for more detailed experimental investigations and species-specific treatment. The variety of diatoms is so large that it is not known whether this asymmetry in timing between the two daughter cells holds for the majority of species or how this time delay can vary.

In both the Müller and Laney models with a delay of one generation, a Fibonacci series for the number of cells replaces the exponential growth. In figure 1*b*, the principle is shown explicitly for the Müller model; in figure 1*c* for the Laney model. Without losses, the number of cells derived from one initial cell follows the series 1, 2, 3, 5, 8, … rather than 1, 2, 4, 8, 16 in the non-delayed scheme. Because of the delay, the division scheme including closure by the sexual phase represents a second-order difference system, but it can be reduced to a first-order matrix equation as shown in electronic supplementary material, S2.

The resulting matrix for the Müller delay model is also non-negative and irreducible, thus the dominant eigenvalue is real positive and given for *α = β* by

3.3 $$\lambda_{\max}=\frac{\beta p}{2}\left(1+\sqrt{1+4\sigma /\beta }\right)$$

(see electronic supplementary material, S2).

That means that, also with this division scheme, the qualitative behaviour does not change, and no true oscillations can be expected. Indeed, simulations with this variant exhibit decaying oscillations similar to those in figure 2 (see electronic supplementary material, S4). The time scale is larger, though, since the vegetative proliferation takes longer to cross all size classes (figure 1*b*). Again, an equilibrium population can be reached only with a special combination of the parameters, such that *λ*_max_ = 1.

In the Laney delay model, the situation is somewhat different. Owing to the transition into a resting phase and back, a short-term oscillation of two generations is induced (for even *n*, this oscillation is sustained; see also electronic supplementary material, S4). In real cultures, it would be difficult to observe such a behaviour, though, because, even under high synchronization, the delay would probably be subject to some distribution. Oscillations of the order of the life cycle decay as in the basic model, with the same time scale as can be deduced also from figure 1*c*. Sustained oscillations have not been found.

As a result from this section, we can conclude that a delay of one daughter cell with respect to further division changes the growth law of the population but does not stabilize oscillations in the life cycle.

### 3.3. Nonlinear processes

In this section, we answer the question of whether nonlinear processes can cause sustained oscillations. In modelling population dynamics, the assumption of limited resources that inhibit the growth of a population is a typical feature for underlying models. But also biparental schemes introduce a second-order nonlinearity by the required meeting probability of the two mating types. For the life cycle scheme of diatoms, we discuss biparental schemes first, and then nutrient saturation. Since linear eigenvalue analysis cannot be applied here, we performed computer simulations for the corresponding models, the *biparental nonlinear model* and the *saturation nonlinear model*.

Biparental reproduction should be modelled as second-order kinetics similar to bimolecular chemical reactions, expressing the dependence on density of the mating strains as reported in the literature [33]. We note, however, that there is a limit even in dense systems since the parent cells can only supply a limited number of gametes. Thus, the number of auxospores cannot surpass the number of parent cells, so there will be a transition to first order with increasing cell number. Modelling a biparental scheme accordingly with the expression for the *biparental nonlinear model* (see Material and methods) results in a slight shift of the targeted equilibrium with time. Simulations showed decaying oscillations similar to the *basic linear model*, but no further stabilization of these oscillations. An illustrating example is given in electronic supplementary material, S4. The positive feedback that is introduced by the nonlinearity (more small cells produce more auxospores than proportionally) obviously is not able to counteract the size broadening mechanism. Sustained oscillations of the life cycle probably cannot be attributed to biparental nonlinearity. For the applicability of the models to different sexual reproduction schemes, however, we can conclude that the linear model is a good approximation also for biparental schemes.

Now, nutrient saturation is discussed. Limitation of nutrients (silica, nitrogen, iron, etc.) would reduce the growth of diatoms if the population is too large and therefore provide a kind of negative feedback. In selecting an appropriate nonlinear expression for saturation, measures have to be taken in a discrete model that the number of individuals will not fall below zero. Therefore, instead of assuming a logistic-type growth model [34], which is the textbook model for discrete difference equations showing oscillations and chaos, we apply the nonlinear model of Ricker [24] and extend it here to our one-species-several-ages case. In the (one-dimensional) Ricker model, the proliferation rate drops exponentially down with the number of individuals as a result of competition for nutrients, but can never become negative as in logistic growth. In our multi-dimensional model, we regard the total number of cells as the limiting factor for saturation, but note that in principle the total surface area or volume could also be considered. Written in terms of our matrix model, this means

3.4 $$x_{t+1}=Px_{t}e^{-\gamma {\left(\sum_{i}x_{i}\right)}_{t}},$$

in which ***P*** is still the linear matrix (*saturation nonlinear model*). The sum in the exponent is over all elements of the population vector, i.e. counting the number of individuals.

In an alternate view of this model variation, we modify *p* into an effective *p*_eff_ depending on the total population

3.5 $$\;p_{\mathrm{eff}}=p_{0}e^{-\gamma \left(\sum_{i}x_{i}\right)}.$$

*γ* is the inverse of the number of cells *K* for which the proliferation rate drops to 1/*e* of the full rate, thus representing an inverse carrying capacity. If we compare this with the one-dimensional Ricker model written as

**Figure 3.**
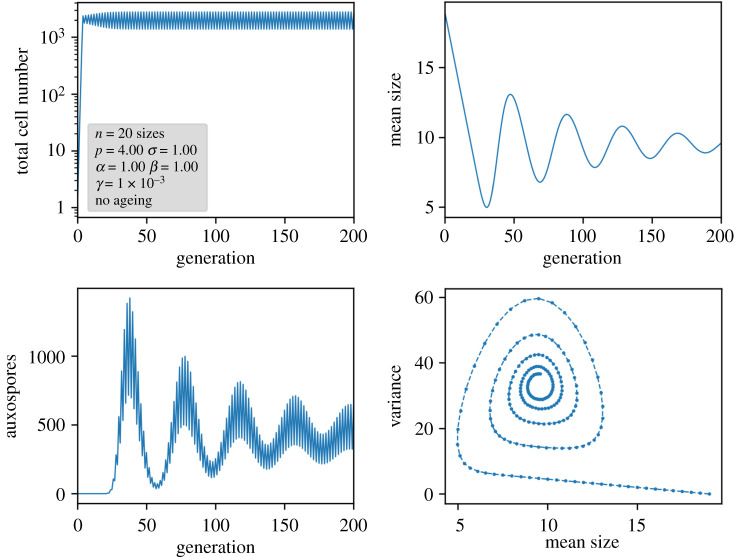
Results of a nonlinear Ricker model with a value for *p* exceeding the critical value for the first bifurcation. It can be seen that the high-frequency oscillation in the population (period 2) does not couple to the generation cycle (period around *n*).

3.6 $$x_{t+1}=x_{t}e^{r}e^{-x/K},$$

with a critical value for the first bifurcation at *r* = 2, it becomes clear that we can expect a similar bifurcation at a critical value of *p*, i.e. *p*_crit_ = *e*^2^/2 ≈ 3.69 (the factor 1/2 arises from the fact that one cell yields two cells in the next generation, thus 2*p* ≡ *e^r^*). Numerical simulations confirm this behaviour and show a decoupling of high-frequency oscillations due to nonlinearity from the cycle through the size classes. Figure 3 shows a representative simulation for *p* = 4. One can see, on the one hand, a persistent high-frequency oscillation in the total number of cells and, on the other hand, the damped oscillation of the mean size and variance leading to a steady-state distribution. A stronger coupling of the nonlinear term to the dynamic size distribution can be achieved by defining the carrying capacity via the total cell surface or volume, but, as respective simulations show, the general behaviour at the end of the simulation does not change. As long as there is an equilibrium distribution as attractor for the trajectory in the linear model, oscillating behaviour is controlled by the value of *p*, up to further bifurcations and eventually chaos. For the Ricker model, however, a *p*_crit_ of 3.69 for the onset of oscillations is too large to be reasonable, since, in the biological model, no cell can produce more than two daughter cells in the next generation (i.e. *p* ≤ 1). It might be possible to redefine the time scale by assigning one discrete step to several generations at the cost of complicated proliferation schemes, but clearly the high-frequency oscillations for large *p*-values are not the oscillations we are looking for. It should be mentioned that we investigated several other nonlinear models such as the three-parameter Hassell model [35], and obtained similar results by detecting persistent oscillations only in the cell numbers, whereas oscillations in the size distribution still decay.

The effect of nonlinearity in providing an effective *p* to limit the total population can be seen best below the bifurcation onset. For instance, with *n* = 5 (for better numerical accuracy), *p* = 1, *α = β* = 0.8*, s =* 0.2, *γ* = 10^−3^, an equilibrium population of 421.85 cells per volume was obtained, giving rise to *p*_eff_ = 0.656, which perfectly fulfils the equilibrium condition

3.7 $$s_{\mathrm{eff}}=\frac{sp_{\mathrm{eff}}}{p}=\sigma^{n}p_{\mathrm{eff}}=\frac{{\left(1-p_{\mathrm{eff}}\beta \right)}^{n}}{\;p_{\mathrm{eff}}^{n-1}},$$

expected for the linear system.

In conclusion for the saturation nonlinearity, we can exclude this effect as the cause for sustained oscillations with periods of the order of the number of generations. The simulations showed clearly a decoupling of population control and size control. This did not change if we include the size in determining the saturation according to

3.8 $$\;p_{\mathrm{eff}}=pe^{-\gamma \left(\sum_{i}x_{i}^{2}\right)}\;(\mathrm{total}\;\mathrm{surface}\;\mathrm{area})$$

or

3.9 $$\;p_{\mathrm{eff}}=pe^{-\gamma \left(\sum_{i}x_{i}^{3}\right)}\;(\mathrm{total}\;\mathrm{cell}\;\mathrm{volume}\;\mathrm{volume}),$$

even if there is an explicit dependence on the size distribution.

### 3.4. Modelling with ageing

In this section, we answer the question of whether limited lifespans of individual thecae can cause sustained oscillations. The problem with all the models discussed up to this point is that a newly generated theca stays in the system and is not removed faster than thecae that are produced later. But in order to maintain a narrow distribution, it is necessary that larger or older cells are removed. Thus, a possible mechanism would be a limited lifespan of an epithecal half. Indeed, there are some experimental reports addressing this issue, such as the one by Jewson [36] for the centric diatom *Stephanodiscus neoastraea.* Jewson [37] deduced a lifespan of six to eight generations and extrapolated a similar value for *Aulacoseira subarctic*a. Laney *et al*. [32] posed the hypothesis that, by the bias to the smaller daughter cell, damaged cell material can be divided asymmetrically, ensuring the quality of inherited material. The other, larger half will accumulate defects and eventually die earlier. This principle of genetic quality control by asymmetric cell divisions and implications for population development is found in several unicellular organisms, for instance in *Escherichia coli* and in *Saccharomyces cerevisiae*, in which the asymmetry in cell division is much more pronounced [31,38]. For the latter, it is known that one single mother cell can only produce 20–25 daughters by budding before dying, which clearly defines a replicative lifespan. Budded daughters always start as juvenile cells independent of the age of the mother cell [39].

Therefore, we applied the *ageing model* in which the age of a cell is defined by the number of cell divisions its epitheca has already undergone. For the sake of simplicity, we define a fixed number of generations a theca may survive. Mathematically, this means that the population is represented by an *n* × *m* matrix in which *n* (index *i*) denotes the number of size classes and *m* (index *j*) defines the lifespan in generations. In order to keep the concept of a distinct lifespan meaningful, ageing for epithecae has to be more pronounced than for hypothecae. In our model, the smaller, ‘younger’ cell keeps its age, whereas the larger, ‘older’ cell ages by one generation. When a cell reaches its lifespan after *m* generations, it is removed from the model.

We note that in the (biologically unrealistic) limiting case that only the hypothecal daughter survives in each cell division (lifespan = one generation), a perfect oscillation would be obtained. In this special case, the size for the single remaining cell would permute through all size classes until sexual reproduction would produce a single new initial cell.

**Figure 4.**
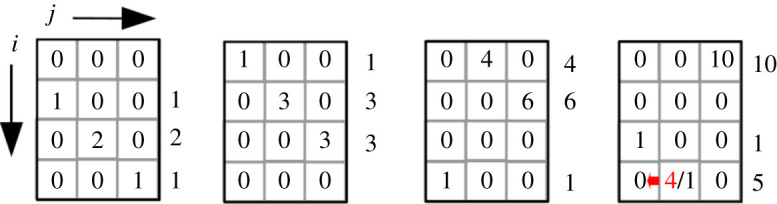
Age treatment of auxospores. *i* represents the size class and *j* the age. They retain their age or are set to pristine again (arrow). The 4 sizes × 3 ages model, starting with the third generation. The narrowing distribution is indicated in the columns to the right of the matrices.

Normally, we would assume that new initial cells are pristine, i.e. we set the age to zero. It is also instructive, however, to consider an alternative case in which new initial cells retain a memory of the age of their parent cells. Such a situation could hypothetically arise in the case of vegetative cell size enlargement in order to start the cycle again. Both possibilities are indicated in a small model system with *n* = 4 and *m* = 3 in figure 4, indicating the very first generations.

Pristine initial cells definitely constitute the biologically more plausible case, so we confine our analytical treatment mainly to this case. We note, however, that, in the case of an age memory upon re-entry of initial cells, the conditions that mathematically prevent sustained oscillations are not fulfilled any more (electronic supplementary material, S3) and the process may become cyclic. Therefore, in the computer simulations, both variations are treated for a comparison of these contrasting behaviours.

In order to analyse the results for arbitrary lifespans mathematically, we rewrote the state matrix as a vector of size *nm* and therefore the propagation as *nm* × *nm* matrix (see Material and methods section). Analytical treatment (electronic supplementary material, S3) leads to the following conclusions about the *nm* eigenvalues, although an analytical expression could not be given: first, 0 is a (*n* − 1)(*m* − 1) fold eigenvalue; second, there is exactly one positive real eigenvalue. Again, for conservation of population number, this eigenvalue can be 1 for an exact combination of *p* (or *p*_eff_ in the case of nonlinearity) and *σ*. Third, there are at most *m* negative real eigenvalues. That leaves a spectrum of minimum *nm* − (*n* − 1)(*m* − 1) − 1 −* m* = *n* − 2 complex eigenvalues.

**Figure 5.**
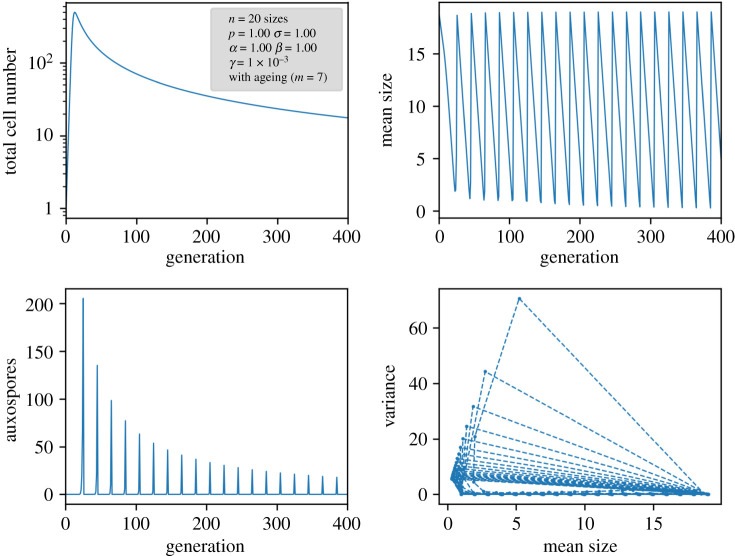
Results of a nonlinear ageing model in which auxospores carry a memory of the previous age.

**Figure 6.**
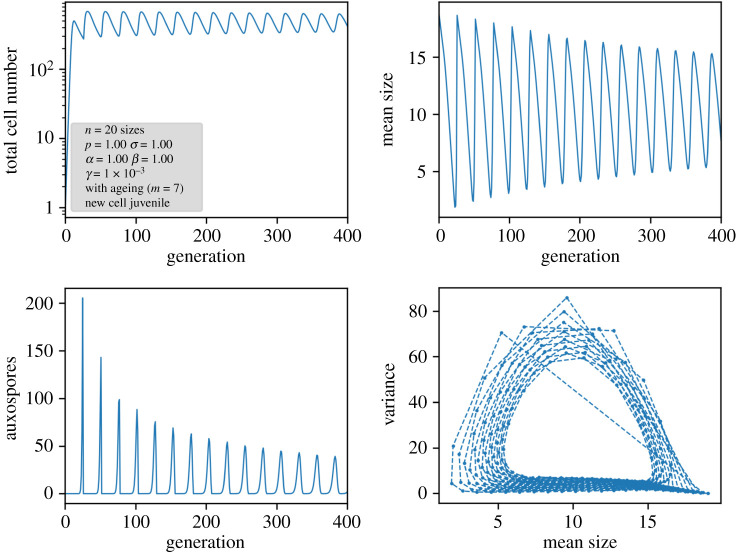
Results of a nonlinear ageing model in which auxospores are always pristine. In the lower right plot, the trajectory has to be read counterclockwise.

In order to see how they contribute to the time development of the population, simulations were performed. Respective results of a larger system are displayed in figure 5 for the variant with age memory of initial cells and in figure 6 for pristine initial cells. In all cases, a nonlinear Ricker term as explained above was implemented to keep an upper limit for the total number of cells.

If the auxospores retain their age, indeed sustained oscillations with a periodicity of the number of size classes are obtained (figure 5). This periodicity is induced by the circular character of the matrix and the constant removal of the generation formed *m* generations before. A closer look at the size distributions reveals that, despite stable oscillations, a proper limit cycle in the sense of a cyclic propagation through a set of defined population states is not followed. Since the population distribution via Pascal's triangle is cut off after *m* values, the population becomes narrower from generation to generation. This can be seen in the variance plot of the simulations and in detail in the small model system of figure 4. The population becomes more and more dominated by the oldest size class. Thus, under the assumption of an ‘age memory’ in the auxospore, an oscillation will not decay and is subject to self-narrowing of the distribution by the MacDonald–Pfitzer process. At the moment, however, there is no hint that this assumption is justified in real diatom species.

A different picture arises if new cells from auxospores are juvenile again. Here, the circular character of the propagation mechanism breaks down, and, in each generation, the re-entry of large, young cells is possible. This leads again to a smearing of the population distribution and results in a damped oscillation towards a steady state (figure 6). Owing to the permanent reset of age, the distribution is smeared again, as indicated by the width of the auxospore peaks. An interesting feature arises also in the periodicity of the oscillations: now they deviate from the number of generations. Instead of the expected periodicity of 20 in figure 6, the periodicity in the mean size, auxospore formation and variance is 26 generations for *n* = 20 and *m* = 7. This periodicity can be explained by considering the offspring of an auxospore until the next pristine auxospore is formed. The population moves diagonally through the *n* × *m* matrix, cells exceeding the right-hand border being extinguished. After *n + m* − 1 generations, all cells derived from a single auxospore have disappeared, with the exception of the newly created auxospores (for details, see electronic supplementary material, S3). This *n + m −* 1 periodicity has been confirmed in numerical simulations by computing the Fourier transform of the oscillations for various values of *n* and *m*. Only for a large *m*/*n* ratio are some small deviations detected which we can attribute to a nonlinear distortion and numerical uncertainties. A selection of simulated data are given in table 2, the full dataset with various variations in the different model parameters is compiled in electronic supplementary material, S6.

**Table 2.** Selection of simulation results under the assumption of a limited lifespan and juvenile initial cells. In all simulations *p* = *α* = *σ* = 1, *γ* = 0.001. The table shows the other parameters, average oscillation period in generations from Fourier transform and fitting parameters for equilibrium distribution and relaxation of population maxima and minima as given in the text.

*n*	*m*	*β*	period	*c*_max_	*τ*_max_	*c*_min_	*τ*_min_	*n*_B_	*a*	*b*
20	5	1	23.7	0.78	1.3 × 10^3^	0.96	2.0 × 10^3^	29.5	2.3	5.9
50	5	1	53.9	0.56	19.0 × 10^3^	0.98	70.8 × 10^3^	147.7	3.7	27.5
70	5	1	73.9	0.50	47.4 × 10^3^	0.98	262.0 × 10^3^	276.3	4.0	45.0
100	5	1	104.0	0.42	111.2 × 10^3^	0.97	1049.6 × 10^3^	531.0	4.3	71.6
140	5	1	144.0	0.36	214.5 × 10^3^	1.02	3826.1 × 10^3^	1342.6	4.7	157.5
50	2	1	51.0	0.72	177.8 × 10^3^	0.99	330.6 × 10^3^	133.3	1.7	8.8
50	4	1	52.9	0.56	26.2 × 10^3^	1.01	102.4 × 10^3^	150.1	3.1	22.9
50	8	1	56.8	0.65	14.9 × 10^3^	0.92	32.8 × 10^3^	127.3	5.3	32.1
50	12	1	60.6	0.80	13.5 × 10^3^	0.88	16.9 × 10^3^	100.5	6.6	27.3
50	20	1	68.2	1.04	10.7 × 10^3^	0.80	6.7 × 10^3^	68.7	6.4	13.0
50	5	1.9	54.0	0.45	15.6 × 10^3^	0.98	125.8 × 10^3^	244.3	4.2	65.8
50	5	1.4	53.9	0.50	17.4 × 10^3^	0.98	96.1 × 10^3^	192.4	4.0	44.2
50	5	0.8	53.9	0.60	19.3 × 10^3^	0.98	57.5 × 10^3^	124.4	3.4	19.6
50	5	0.5	53.8	0.71	18.7 × 10^3^	0.98	36.3 × 10^3^	89.1	2.8	9.2
50	5	0.2	53.5	1.01	14.9 × 10^3^	0.93	13.4 × 10^3^	58.8	1.5	2.2

In all simulations with pristine auxospores, the oscillations decay towards an equilibrium state, but on a large time scale. Before discussing the interesting details of the long-term relaxation process, we will have a look at the equilibrium state, taken at the end of the simulations (after several million generations).

**Figure 7.**
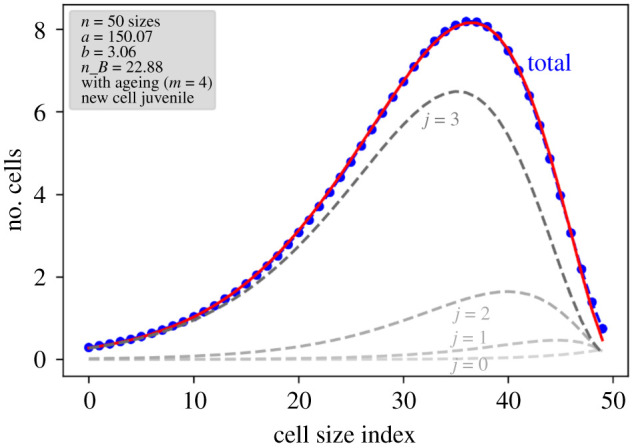
Equilibrium distribution after 5.3 mio. generations for distinct age classes and summed over all ages, with corresponding β-binomial fitting functions. Simulation parameters are *n* = 50, *m* = 4, *p* = *α* = *β* = *σ* = 1, *γ* = 0.001.

The presence of a defined equilibrium is ensured by the nonlinear Ricker term, which controls the total population. From the structure of the *nm × nm* matrix that represents the linear operation on the population matrix, it can be shown that again only certain combinations of *p—*together with the nonlinear Ricker term as *p*_eff_—and *σ* lead to an eigenvalue of 1, which means a stable population. The *n × m* matrix characterizing the population eigenstate expresses a distinct size and age distribution in each age class. Comparable with observables, however, are mainly the cumulated size distributions, summed over the age structure (figure 7). It turns out that the total distribution has a maximum at a specific size class. Age-resolved plots show that this distribution is dominated by the *oldest* cells. For younger cells, the distribution is shifted towards larger sizes, which may be counterintuitive at first sight. However, considering the fact that older cells on average went through more size-reducing cell divisions, one can understand the reason behind this. In figure 4, where the size distributions for different ages can be read as columns, the tendency can already be seen after a few generations.

Fitting attempts with several distribution functions showed that this can be represented empirically by a β-binomial distribution on an inverted size scale, i.e.

3.10 $$\;f(i)=\left(\begin{array}{c} n_{B}\\ n-i \end{array}\right)\frac{B(a+n-i,\;n_{B}+b-n+i)}{B(a,b)}(1-d)+d,$$

in which *i* is again the size index and *B*(*a*,*b*) is the *β* distribution with shape parameters *a* and *b*. In probability distributions, *n*_B_ would represent the upper limit, hence *n*, but here it can be regarded just as an additional fit parameter deviating from *n* and taking also non-natural values. *d* is an offset with renormalization and is negligible in most cases. The distribution function can thus only be taken as an empirical function without some statistical foundation, but it describes the simulated data astonishingly well. Note that this equilibrium distribution differs substantially from the flat distribution function in the simple models. The fitted parameter data are included in table 1. The maximum of the age-cumulated size distribution, calculated as the percentage relative to the largest size, shifts towards larger cells for increasing *n* at constant *m* and towards smaller cells for increasing *m* at constant *n*. The width of the distribution, measured as full width at half maximum, decreases in both cases.

**Figure 8.**
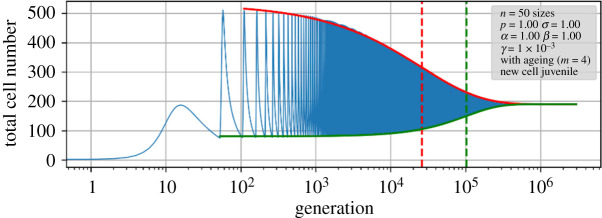
Relaxation of the total cell number towards the equilibrium. Indicated are the enveloping stretched exponential fits for maxima and minima (thick solid lines) with the location of the inflection points (dashed lines). Note the logarithmic time scale. Simulation parameters are the same as in figure 7.

Now we consider the relaxation process from an initial condition towards this equilibrium. For this purpose, we plotted the maxima as well as the minima of the total cell number in each oscillation versus generation *t*. The decline does not follow a simple exponential law—which is understandable considering the nonlinear term and the existence of several complex eigenvalues—but can be reasonably well described by a stretched exponential function, in mathematics also known as the complementary cumulative Weibull function,

3.11 $$N=A_{2}+(A_{1}-A_{2})\exp(-{\left(t/\tau \right)}^{c}),$$

in which *A*_2_ denotes the equilibrium cell number, (*A*_1_ – *A*_2_) is the starting amplitude, *τ* is the scale parameter and *c* is the shape parameter. The scale parameter can be taken as the measure of the mean decay time and is represented graphically as the inflection point when the generation is plotted on a logarithmic time scale (figure 8).

Fitted values describing the decline functions for various model parameters are also compiled in table 1, with the full set in the electronic supplementary material. The following general trends can be extracted from the fits: for increasing the number of size classes *n*, other parameters being constant, the equilibrium population decreases almost exponentially, i.e. with positive curvature, the scale parameter *τ* increases with positive curvature. Interestingly, the shape parameter *c* decreases steadily for the maxima whereas it does not vary so much for the minima and passes through a transient maximum. For increasing lifespan *m*, other parameters being constant, the equilibrium population increases and the scale parameter decreases. The shape parameter increases for the maxima, but decreases for the minima. The higher the preference for younger daughter cells, i.e. for decreasing values of *β*, the lower the population and the scale parameter in the minima. The shape parameter for the decay in the maxima becomes higher. Interestingly, the scale parameter of the maxima and the shape parameter of the minima are maximized for certain values of *β*. Finally, a change in *σ* leads to modifications in the decay parameters for maxima and minima in opposite directions, respectively. In summary, the relaxation parameters have a complex dependence of the model parameters, but long-lasting relaxations (i.e. large-scale parameters) are obtained for large *n*, small *m* and high values of *β*.

Even if an equilibrium point exists, it may never be reached in a real diatom population in a reasonable amount of time. We note that the decay by persistent oscillations comprises many orders of magnitude, of the order of 10^4^–10^5^ generations in the maxima and even more in the minima. This is much longer than any biological system can stay undisturbed by external influences, so, for any practical reason, the oscillation can be regarded as a persistent one. Any perturbation that changes the parameters for vegetative or sexual reproduction slightly would induce a new deflection from equilibrium and start the oscillation anew. Possible factors that may contribute in natural environments are light conditions, temperature, predator occurrence and nutrient availability.

### 3.5. Periodic environmental influences

Fluctuating external factors can be of a periodic nature, typically following the seasons throughout a year. Photoperiod, temperature and nutrients change within a year and favour or disfavour the total growth of a diatom population. Sexual reproduction may be limited to only a few weeks in a year [15]. It can, therefore, be inferred that seasonal changes of environmental factors act as zeitgeber and are coupled to the inherent mechanism of the life cycle by varying the reproduction parameters. This can be expressed as a non-autonomous system in which *p* or *s* is a periodic function of time. We investigate the influence of such an external zeitgeber on the oscillations of the previous model. As the basic mode, we assume a sinusoidal variation and note that other annual variations can be expressed as the sum of the basic mode and its higher harmonics. Usually, a year is shorter than the complete life cycle and can be expressed by *z < n* generations.

**Figure 9.**
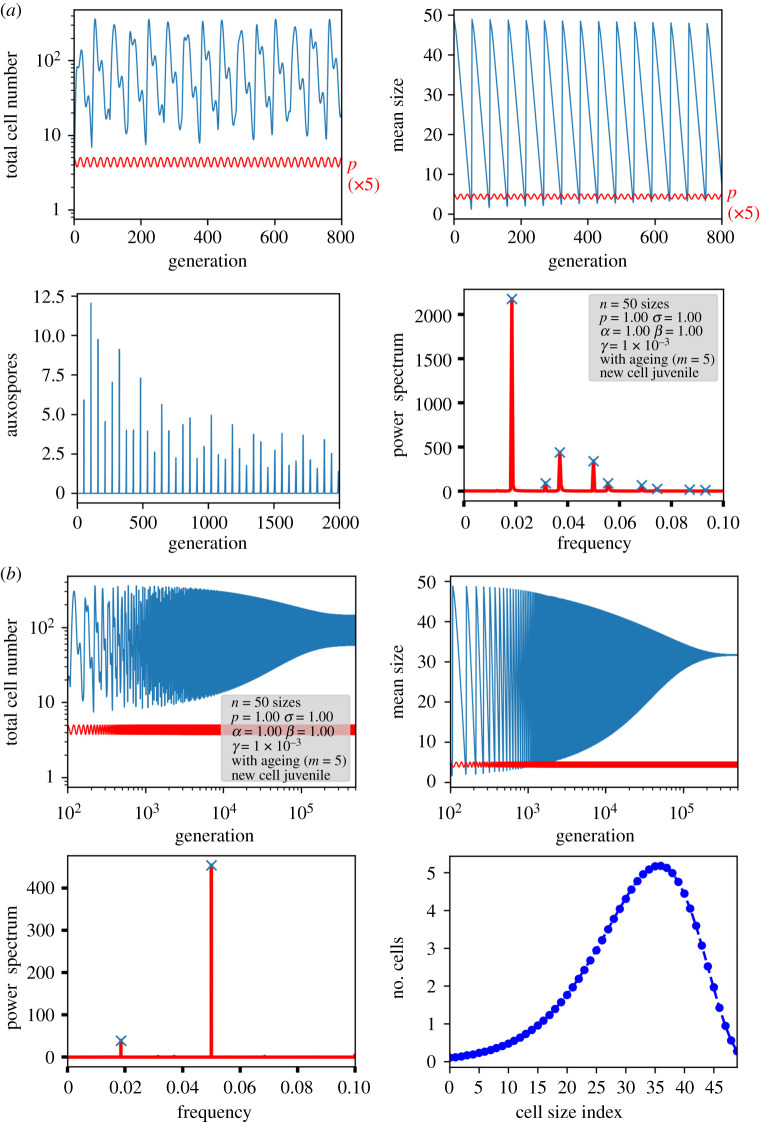
Simulation results for a system with sinusoidal variation of *p* down to 75% of its value, representing an annual zeitgeber. The nominal generation cycle (number of size classes) is *n* = 50 generations, the zeitgeber cycle *z* = 20 generations. (*a*) Behaviour at the beginning. Given is the total cell number, mean size, the auxospore number and the power spectrum of the total cell number. In both upper graphs, the sinusoidally varying value of *p* multiplied by a factor of 5 for better visibility is indicated as the bottom line in red. (*b*) Long-term behaviour. Replacing auxospore number, the size distribution after 10^6^ generations is displayed.

In figure 9*a*, the results of a computer simulation with the *p-zeitgeber model* are shown for a variation of *p* between the original value and 75% of that value in 1 year consisting of *z* = 20 generations, with *n* = 50 size classes. The oscillating behaviour seems complicated, especially in the seemingly random auxosporulation events, but Fourier analysis reveals the occurrence of the *n + m* − 1 generation period of 54 generations (frequency 0.0185) as well as the seasonal period of 20 generations (frequency 0.05). Interestingly, besides the usual higher harmonics, new periodicities arise: the difference frequency at 0.0315 and the sum frequency at 0.0685, which can be attributed to nonlinear coupling of the two periodic processes, since the Ricker term is still included. Like nonlinear saturation, the seasonal change of *p* couples only to the cell number, but not to the size distribution. If the simulation is performed further until 10^6^ generations (figure 9*b*), oscillations in the total cell number remain, but have been decayed for the mean size, leading to an equilibrium size distribution as in the other models. The only remaining Fourier components for the cell number are the 20 generation period, now being dominant, and the 54 (*n* + *m* − 1) generation period.

**Figure 10.**
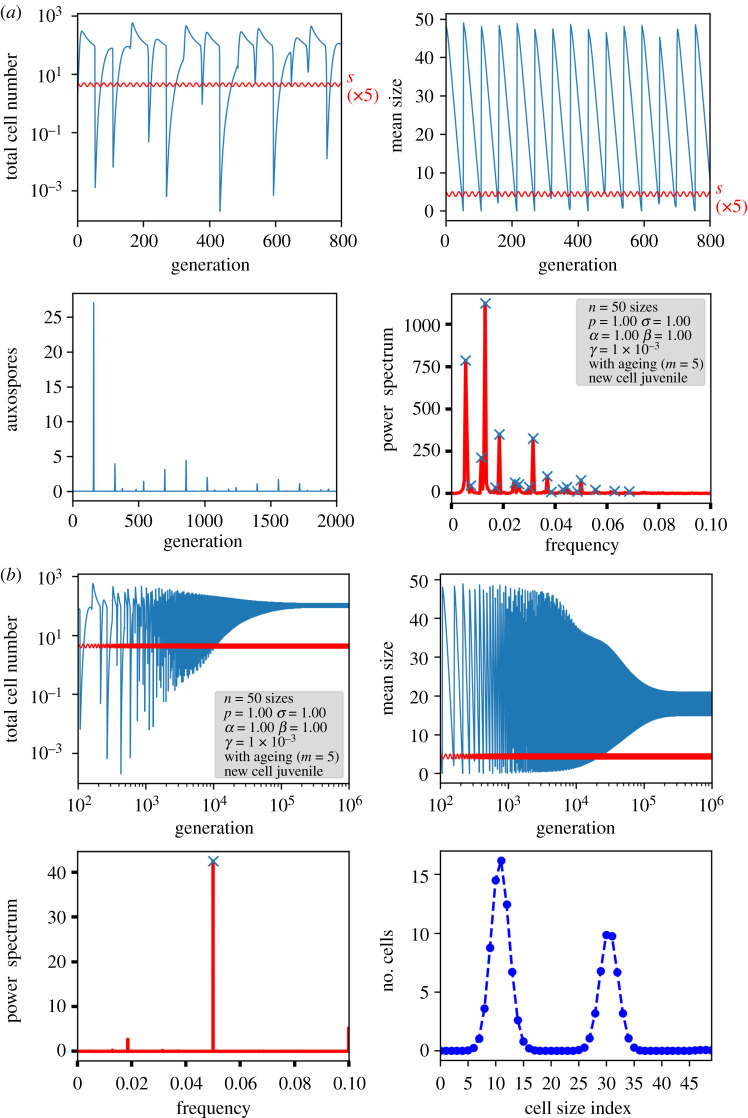
Simulation results for a system with sinusoidal variation of *s* down to 75% of its value. Graphs are displayed as in figure 9 at the beginning (*a*) and for 10^6^ generations (*b*).

The situation is different if, instead of *p*, *s* is varied since the variation concerns only the sexual part, i.e. the population of only the initial cells. In figure 10, the results are displayed for the same parameter set as in figure 9, but with a variation of *σ* between the original value and 75% of that value (*s-zeitgeber model*). Since *s* = *pσ^n^*, *s* drops down to virtually zero during the course of the year. Auxospore formation depends strongly on the phase relationship between total cell number and external signal, so there are some years in which there are almost no auxospores. The pattern in the frequency space at the beginning is much more complicated, with low-frequency components indicating periodicities with long time scales, much longer than the generation period. After 10^6^ generations, again the annual period dominates the population, but there is still an oscillation in the mean size. The size distribution reveals the presence of a typical multimodal distribution (figure 10*b*, lower right), in which each peak can be attributed to a single year. These peaks traverse the size distribution, stabilizing the *n* + *m* − 1 generation period. Here, we can see clearly that a variation in *s* directly influences the cell size distribution and, therefore, keeps it oscillating, whereas a variation in *p* (figure 9) only influences the total cell number and, therefore, leads to the original equilibrium distribution. If there is no ageing, a multimodal distribution still exists, but is less narrow. The peak in the power spectrum for the *n* + *m* − 1 vanishes, which means that thev zeitgeber signal defines the only remaining time scale (see electronic supplementary material, S4e).

We can state as result of this section that seasonally varying sexual reproduction behaviour may supply the necessary driver for keeping the generation clock running. Without ageing, however, the annual zeitgeber clock overwrites this clock completely and imprints its own rhythm.

## 4. Conclusion

Is the life cycle of diatoms a clock? If we mean limit cycles that consist of a series of defined population states that are adopted sequentially and return exactly back to previous states, then, at the moment, we would have to answer *no* with the present knowledge about the proliferation mechanism. The problem is the size distribution that needs some self-narrowing mechanism in order to compensate broadening exactly. If we assume finite lifetimes for a newly formed theca, then older, larger cells are removed effectively and counteract the broadening. With the additional assumption of age memory in auxospores, self-sustained rhythms with even further size narrowing can occur. But also without this somewhat arbitrary additional assumption, the oscillatory decay towards an equilibrium is significantly slowed down by the proposed finite lifetimes and takes place on a time scale of millions of generations, enough to keep the oscillations running by statistical fluctuations. If we define a biological clock in the common sense as ‘an inherent timing mechanism in a living system that is inferred to exist in order to explain the timing or periodicity of various behaviours and physiological states and processes' [40], then the answer to our question is *yes*, but it resembles more a cuckoo clock that has to be wound up from time to time. This could happen by environmental fluctuations, ranging from singular environmental events bringing the size distribution again away from equilibrium up to the annual period of the sexual phase.

The results presented here may help in guiding experimental investigations of previously unknown or even contradictory data connecting cell cycle and life cycle. If, for instance, more data about the lifespan of a single theca and the asymmetry in timing between the daughters were known, population dynamics of diatoms could be understood better. This may turn out to be useful—together with knowledge about nutrient supply currents—for modelling algal blooms and periodic events in the carbon cycle.

## Data accessibility

All data including program code are given in the electronic supplementary material.
